# Complete remission with olaparib in *BRIP1*-mutated metastatic high-grade pleomorphic sarcoma: case study and literature review – an example of a genomic profiling-based tumor treatment, in a cancer type with high unmet clinical need

**DOI:** 10.2340/1651-226X.2025.43374

**Published:** 2025-09-23

**Authors:** Alexander D. J. Thooft, Sofie Joris, Celine Jacobs, David Creytens, Sylvie Rottey, Brigitte Maes, Philippe Aftimos, Lore Lapeire

**Affiliations:** a Department of Medical Oncology, Ghent University Hospital, Ghent, Belgium; b Department of Internal Medicine and Pediatrics, Faculty of Medicine and Health Sciences, Ghent University, Ghent, Belgium; c Department of Medical Oncology, University Hospital Brussels, Brussels, Belgium; d Department of Pathology, Ghent University Hospital, Ghent University, Ghent, Belgium; e Laboratory for Molecular Diagnostics, Jessa Hospital, Hasselt, Belgium; f Faculty of Medicine and Life Sciences, LCRC, University of Hasselt, Hasselt, Belgium; g Clinical Trials Conduct Unit, Institut Jules Bordet – Hôpital Universitaire de Bruxelles, Brussels, Belgium

**Keywords:** poly(ADP-ribose) polymerase inhibitors, BRIP1 protein, human, sarcoma, recombinational DNA repair, remission induction, high-throughput nucleotide sequencing, precision medicine

## Abstract

**Background and purpose:**

Patients with high-grade metastatic sarcoma have a poor prognosis and limi-ted treatment options, mostly involving chemotherapy with palliative intent. In the past years, next generation sequencing has proven its benefit in cancer diagnostics and prediction of treatment response to targeted therapy.

**Patient/material and methods:**

We present a case of response and long-term complete remission under treatment with the poly(ADP-ribose) polymerase inhibitor (PARP-inhibitor) olaparib in a patient with meta-static high-grade pleomorphic sarcoma, with an next generation sequencing detected *BRIP1*-mutation. Additionally, a literature search regarding the pathophysiology of *BRIP1*-mutations and the role of PARP-inhibitors in *BRIP1*-mutated cancer was conducted.

**Results:**

A 67-year-old female patient was diagnosed with a high-grade intra-abdominal pleo-morphic sarcoma, which was surgically resected. One year later, metastatic lesions in the right lung were observed. Genomic profiling identified a *BRIP1*-mutation. Based on this finding, the patient was included in the PRECISION-2 olaparib study, which evaluates the efficacy of olaparib in advanced cancers of any type harboring mutations in a homologous recombination gene. Within 2 months of ola-parib treatment, regression of the pulmonary metastases was observed with ongoing complete remission for currently 36 months. A review of the available literature highlights the importance of *BRIP1* in the homologous recombination repair pathway and its role as a cancer susceptibility gene. Studies in *BRIP1*-mutated breast cancer, ovarian cancer, and prostate cancer suggest a clinical benefit of PARP-inhibitor use.

**Interpretation:**

We here describe the first case of a metastatic *BRIP1-*mutated sarcoma, undergoing a complete radiologic response to olaparib treatment. We highlight an underexplored role of homologous recombination deficiency in non-traditional cancer types and postulate a tumor-agnostic approach to the use of PARP-inhibitors in *BRIP1*-mutated tumors.

## Introduction

Soft-tissue sarcomas (STSs) are a heterogeneous group of mesen-chymal tumors with locally destructive growth and high risk of both local and distant recurrences.

The standard of care treatment for high-grade STS in adult patients is a wide surgical excision with R0-margins often followed by adjuvant radiotherapy (RT) and in selected cases adjuvant chemotherapy [1]. Neoadjuvant treatment in the form of chemotherapy or RT can be considered in case of technically unresectable/borderline resectable tumors [2]. In oligometastatic setting, local treatment such as a pulmonary wedge resection, stereoablative RT, or interventional techniques (radiofrequency- or cryoablation) can be considered [2].

Unresectable metastatic disease is treated with chemotherapy, most commonly with an anthracycline-based therapy. For doxorubicin at a dosing schedule of 75 mg/m^2^ every 3 weeks, the overall response rate is 10–25%, and the stable disease rate is 20–40% [3]. The median progression-free survival (PFS) and overall survival (OS) for doxorubicin are 4–5 months and 8–14 months, respectively [3]. Combination therapy on an anthracycline backbone has a higher response rate but is associated with greater toxicity without OS benefit [3]. With doxorubicin, there is a dose limiting cardiotoxicity risk, and a maximum lifetime cumulative dose of 450–550 mg/m^2^ needs to be respected. Therefore, patients can only safely receive up to six cycles of doxorubicin [4].

Second-line chemotherapy regimes may include gemcitabine-docetaxel, trabectedin, and pazopanib [3].

During the course of systemic treatment, surgery or RT of responding metastases may be considered taking into account the site, tumor extent, and natural history of the disease in the individual patient [1, 2, 5].

Treatment of metastatic high-grade STS in adults remains highly challenging, as the prognosis is poor with an overall survival of 5% at 5 years [6, 7] and a median survival of approximately 12–19 months from the onset of metastatic disease [5, 8]. This stresses the importance of further research on additional treatment options. One potential route of research involves the role of homologous recombination deficiency (HRD).

## Material and methods

We present a case of complete remission under treatment with olaparib in a patient with metastatic high-grade pleomorphic sarcoma with a germline *BRIP1*-mutation. All information in the case report was – with consent of the patient – extracted from the electronic patient record management system used at Ghent University Hospital, Belgium. The case report was written in accordance with the Consensus-based Clinical Case Reporting (CARE) guidelines. Figures were created using either Microsoft PowerPoint or GIMP 2.10.38.

Additionally, an extensive review was performed of the available literature on the pathophysiology of *BRIP1-*mutations, the use of poly(ADP-ribose) polymerase inhibitors (PARP-inhibitors) in *BRIP1*-mutated ovarian, breast, prostate, and pancreatic cancer, as well as the use of PARP-inhibitors in STS and other cancer types. Several search engines (including but not limited to PubMed, Google Scholar, and Embase) were employed. Used entry terms included *BRIP1*, *BAHC1*, *FANCJ*, Fanconi anemia, Poly(ADP-ribose) Polymerase Inhibitors, Olaparib, Talazoparib, Rucaparib, Niraparib, HRD, Soft-tissue sarcoma, Pleomorphic sarcoma, Ovarian cancer, Breast cancer, Prostate cancer, Pancreatic Cancer, Comprehensive genomic Profiling (CGP), and Precision medicine.

As high-grade pleomorphic sarcoma is a rare cancer type, and the available data on the use of PARP-inhibitor in *BRIP1-*mutated tumors stem mainly from case reports, we performed a narrative review as opposed to a systematic review, as this seemed the best way to summarize the limited available literature.

## Results

### Case description

**Figure 1:**
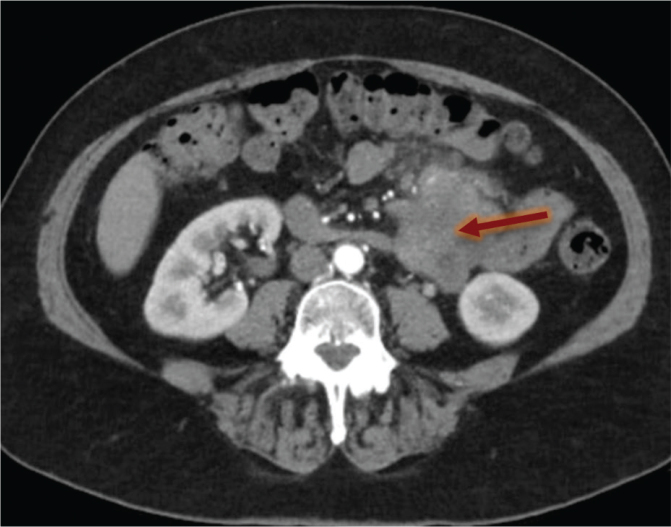
The undifferentiated pleomorphic sarcoma at diagnosis (indicated by an arrow).

In March 2021, a 67-year-old female patient with no relevant medical history but with a family history of early-onset breast cancer (sister at the age of 37) was diagnosed with an undifferentiated pleomorphic sarcoma presenting as a large tumoral mass in the mesentery of the small intestine at the Angle of Treitz (Figure 1). No metastases were visualized on imaging. Surgical removal of the tumoral mass with a maximal diameter of 10 cm was performed. Histology showed spindle-shaped and polygonal poorly differentiated tumor cells with marked pleomorphism, arranged in a haphazard to fascicular growth pattern. Large, bizarre multinucleated cells and numerous mitotic figures were identified. The tumor cells lacked any evidence of epithelial differentiation. Immunohistochemistry showed absence of specific line of differentiation: the tumor cells stained positive for Vimentin but displayed no expression of epithelial markers (Cytokeratin MNF116, Cytokeratin 8/18, Cytokeratin 5, Cytokeratin 7, Cytokeratin 20, and EMA), melanocytic markers (S100 and SOX10), myogenic markers (SMA and Desmin), vascular markers (CD34 and ERG), lymphoid markers (CD45, CD3, CD20, CD5, and CD21), lipogenic markers (MDM2), or ‘gastrointestinal stromal tumor’ markers (CD117 and DOG1). The tumor cells showed a preserved expression of SMARCB1 (INI1) and SMARCA4. The tumor margins were negative, and the resected lymph nodes had no tumoral invasion. As such, the pathology report was consistent with a high-grade pleomorphic sarcoma, pT2N0M0, FNCLCC grade 3, R0-resection. The patient received no neoadjuvant or adjuvant treatment.

**Figure 2:**
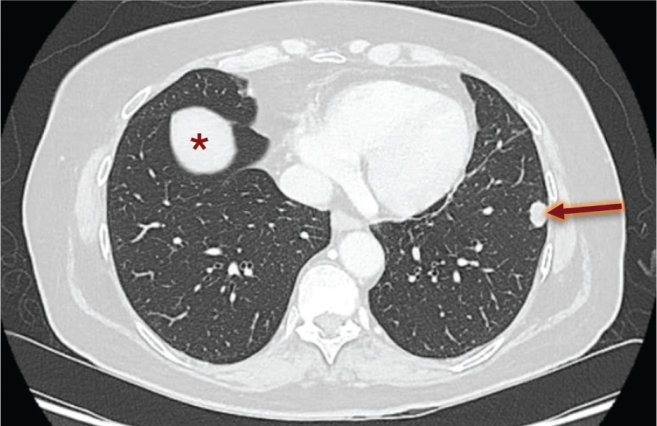
Single lung metastasis before wedge resection (indicated by an arrow). *Axial liver cross-section showing on CT-scan of thorax; no metastatic lesion.

**Figure 3:**
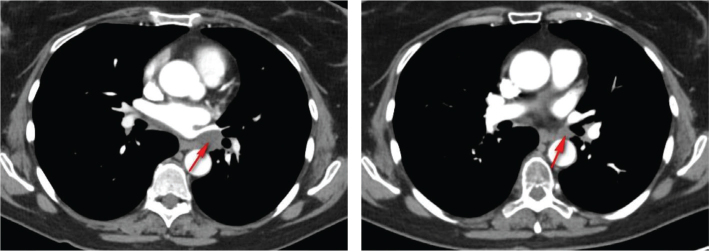
Left hilar lymphadenopathy before and after irradiation (indicated by an arrow).

In July 2021, a single tumoral lesion in the left lung was identified (Figure 2) and resected. Histopathological analysis confirmed the hypothesis of a metastasis of the high-grade pleomorphic sarcoma. In January 2022, a left hilar lymphadenopathy (as shown in Figure 3) was visualized and confirmed to be infiltrated by sarcoma cells via endobronchial ultrasound (EBUS) diagnostic function. Stereotactic RT (18 daily fractions of 3 Gy) of this lesion was performed. Unfortunately, in May 2022, multiple asymptomatic metastatic locations were visualized in the right lung, signaling the need for a systemic therapy. In discussion with the patient, no immediate systemic therapy was started, and options for study inclusion were first explored. The patient consented to participate in the Belgian Approach for Local Laboratory Extensive Tumor Testing or BALLETT study (NCT05058937) [9]. The main objective of the BALLETT study was to offer comprehensive genomic profiling (CGP)-guided treatment recommendations fostering precision oncology. For this CGP, the Illumina trusight oncology 500 assay was used. Recommendations were made by the National Molecular Tumor Board established within the Belgian Society of Medical Oncology (BSMO)-PRECISION framework. Treatment recommendations could consist of advice for approved drug use, to participate in a clinical trial or a medical need program, or for off-label drug use and included advices for both molecular guided therapy and/or immunotherapy.

**Figure 4:**
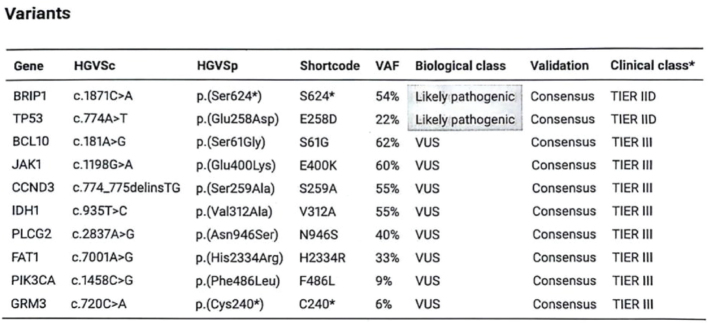
The patient’s comprehensive genomic profiling results for the BALLETT study.

The earlier resected lung metastasis was sent for CGP analysis, which revealed a pathogenic *BRIP1*-mutation (*BRIP1* c.1871C>A p.(Ser624*)) enabling enrollment in the PRECISION-2 olaparib study (NCT03967938). The PRECISION-2 olaparib study [10, 11] intents to evaluate the efficacy of olaparib in advanced cancers of any type occurring in patients with germline or somatic tumor mutations in one of the evaluated homologous recombination repair genes. The results of the performed CGP are depicted in Figure 4. More recently, performed germline testing was also positive for the same *BRIP1*-mutation.

**Figure 5:**
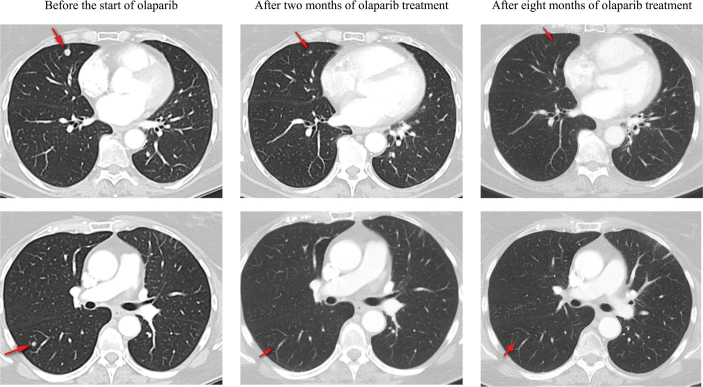
Two examples of complete oncological response of lung metastases after olaparib treatment (indicated by arrows).

Olaparib at a dose of 300 mg bi-daily (BID) was commenced as a first-line treatment in July 2022. Imaging studies after 2 months on olaparib showed partial regression of the known lung metastases. Follow-up scans were repeated every 2–3 months, and complete oncological response was noted 8 months after olaparib initiation. In Figure 5, radiographic responses of two lung lesions are illustrated. Therapy was well-tolerated with no major side effects and no need for dose reductions.

At present, 36 months after the initiation of olaparib, the patient is still in sustained oncological complete remission with good quality of life in a chemotherapy-free treatment regimen.

### Literature review

#### Homologous recombination deficiency

Human DNA takes the form of a double helix. During replication, both single-strand (ssDNA) and double-strand (dsDNA) DNA breaks can occur [12]. Homologous recombination (HR) ensures error-free repair of dsDNA breaks [13].

Both germline and somatic mutations in genes involved in the HR-cascade can, however, impede a cell’s ability to effectively repair these dsDNA breaks [12]. This phenomenon is known as HRD and has proved to be a key player in the development of certain cancer types. In the early 1990s, both *BRCA1* [14] and *BRCA2* [15] were identified as cancer susceptibility genes in patients with early-onset breast and ovarian cancer. Since then, additional genes involved in the HR-pathway, such as *PALB2*, *ATM*, and *CHECK2* [16], have been identified, and a clear link between HRD and breast, ovarian, prostate, and pancreatic cancer has been established. A somewhat newer entity is *BRIP1*, which exerts its function on a cellular level in conjunction with *BRCA1* and has likewise been identified as a cancer susceptibility gene [13, 17, 18].

**Figure 6:**
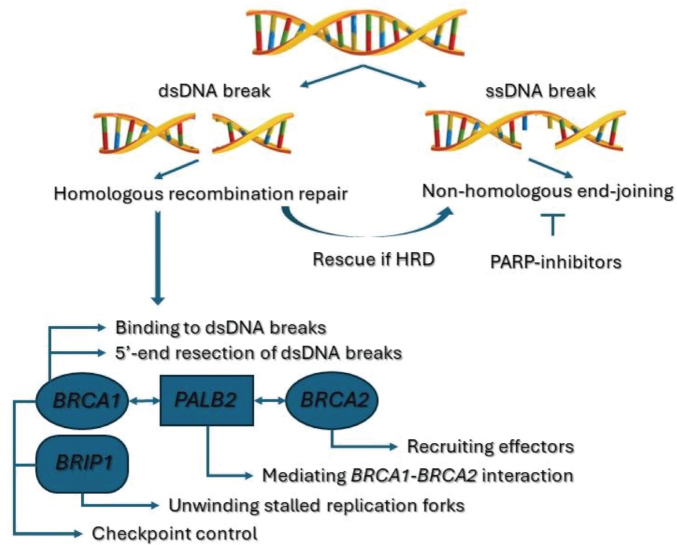
Role of *BRCA1, BRCA2, PALB2*, and *BRIP1* in the homologous recombination pathway.

An overview of the HR-pathway and the role of *BRIP1* are presented in Figure 6. A more in depth description is presented in Supplementary Appendix 1.

Initially, the identification of germline HRD-mutations enabled better cancer screening strategies for patients and their relatives. Moreover, better advice on preventive measures including surgical removal of breasts and ovaries and reproductive genetic counseling could be provided to individuals with a germline HRD-mutation [19]. With a growing understanding of DNA repair pathways, the identification of both germline and somatic loss-of-function mutations in HR-genes also became of therapeutic significance. These mutations can sensitize tumors to cross-linking agents such as platinum-based chemotherapy, causing dsDNA breaks and to PARP-inhibitors, inhibiting ssDNA break repair and hereby inducing synthetic lethality [12, 17]. As of today, the use of four PARP-inhibitors is approved by the *European Medicines Agency*. Indications [20–23] are listed in Supplementary Appendix 2.

#### BRIP1 as a cancer susceptibility gene

Considering the presumed role of *BRIP1* in the HR-cascade, studies have explored the significance of *BRIP1* as a cancer susceptibility gene. Loss-of-function mutations in *BRIP1* resulting in reduced helicase activity and/or altered *BRIP1-BRCA1* interaction [17] have been linked to an increased risk for breast [17, 19, 24], ovarian [19, 25–27], prostate [28, 29], and pancreatic cancer, [30, 31]. Furthermore, it is noteworthy that bi-allelic inheritance of mutations in *BRIP1*, as well as *BRCA2* and *PALB2*, contributes to the rare disease of Fanconi anemia, with patients being prone to both hematological and solid cancer development [17, 32].

#### BRIP1-mutations as predictive markers for PARP-inhibitor use in breast, prostate, ovarian, and pancreatic cancers

Limited but growing evidence suggests a potential clinical benefit of PARP-inhibitors in patients with one of the classical HRD-associated cancer types, in whom a *BRIP1*-mutation has been identified.

Trials evaluating the benefit of PARP-inhibitors in breast cancer patients with mutations in HR-pathway genes other than *BRCA1/2* have only included a very limited number of *BRIP1*-mutation patients and have not been able to confirm a PARP-inhibitor benefit for *BRIP1*-carriers [33, 34]. A case presented by Kwapisz et al. [35] of a patient with *BRIP1*-mutated metastatic hormone receptor positive breast cancer demonstrated, however, a resolution of symptoms, significant decrease in CA15.3, and complete metabolic response on positron emission tomography-computed tomography scan after 8 months of olaparib treatment.

In both the TRITON2 [36] and PROfound trial [37] signs of a PFS benefit of PARP-inhibitor use in metastatic castration resistant prostate cancer patients with *BRIP1*-mutations were noted. In the TRITON trial, one of two included patients with a *BRIP1*-mutation had a radiographic and PSA response to rucaparib. The PROfound trial included patients with either a *BRCA1/2, ATM, BRIP1, BARD1, CDK12, CHEK1, CHEK2, FANCL, PALB2, PPP2R2A, RAD51B, RAD51C, RAD51D*, or *RAD54L* mutation in the tumor tissue and compared the use of olaparib versus a control group receiving enzalutamide or abiraterone + prednisone. The median imaging-based PFS was significantly longer in the olaparib group than in the control group (5.8 months versus 3.5 months). No separate outcome data of the 4 enrolled patients with a *BRIP1*-mutation were published [37].

The clinical benefit of PARP-inhibitors in ovarian cancer has been clearly established [38–41]. We identified no studies specifically focusing on the benefit of PARP-inhibitors in *BRIP1*-mutated ovarian cancer. However, aside from *BRCA* status, platinum sensitivity and genomic instability are associated with a better response to PARP-inhibition [42]. As *BRIP1*-mutations are both linked to platinum sensitivity [43, 44] and genomic instability [17], clinical benefit of PARP-inhibitor use is to be expected. Moreover, the presence of a pathogenic *BRIP1*-mutation will often result in a positive HRD-testing, therefore qualifying the patient for olaparib (cfr. Appendix 2) [45]. Consequently, most patients with *BRIP1-*mutated platinum-sensitive stage III or stage IV high-grade ovarian cancer could already receive PARP-inhibitor maintenance therapy.

Finally in pancreatic cancer, trials with niraparib and rucaparib in patients with HRD-mutations other than *BRCA1/2*, including *BRIP1*, are ongoing [46].

Currently, studies are exploring the potential impact of different types of *BRCA1/2* mutations on PARP-inhibitor efficacy. A varying degree of PARP-inhibitor benefit according to the *BRCA1/2* mutation type and site is seen [47, 48]. In *BRIP1*, different loss-of-function mutations may have a potential impact on PARP-inhibitor response as well. Literature on this topic is, however, very limited and inconclusive [49, 50]. The patient in the presented case has a pathogenic *BRIP1* c.1871C>A p.(Ser624*) mutation. This mutation changes the amino acid from a serine to a stop codon within coding exon 12 and is expected to result in loss of function by premature protein truncation (ClinVar version 2025-06-30, RCV000129060). This specific alteration has been reported before in 2 ovarian cancer cases [51, 52] and 2 cases of breast cancer [53].

#### BRIP1- and other HRD-mutations as predictive markers for the use of PARP-inhibitor in other malignancies

As stated earlier, HR-mutations have been linked to a specific subset of cancers, primarily consisting of breast, ovarian, prostate, and pancreatic cancers. Newer insights are, however, indicative for a broader role of HRD in other cancer types [11, 54, 55].

#### PARP-inhibitors in HRD-mutated endometrial cancer

The use of PARP-inhibitors is gaining more interest in endometrial cancer (EC).

Recent studies have shown improvements in disease-free and overall survival in advanced and metastatic EC by adding immunotherapy to chemotherapy [56]. Both the RUBY part 2 [57] and DUO-E [58] trials are investigating whether the addition of, respectively, niraparib and olaparib to the maintenance immunotherapy could further benefit survival. It is important to note that in both trials, no direct head-to-head comparison between the chemotherapy plus immunotherapy arm and the chemotherapy plus immunotherapy plus PARP-inhibitor arm was made; both arms were compared to the chemotherapy only control arm. Therefore, only exploratory subgroup analyses of the added value of PARP-inhibitors can made. One of these subgroup analyses of the DUO-E trial suggested a further benefit with the addition of olaparib to durvalumab in the pMMR subgroup but not in the dMMR subgroup [58].

The UTOLA trial [59] evaluated the effectiveness of olaparib maintenance in patients with advanced EC, who achieved disease control after first-line platinum-based chemotherapy. Olaparib did not improve PFS compared to placebo in all participants, but in HRD-tumors, the median PFS was statistically higher with olaparib: 5.4 (90% CI, 3.6–9.6) versus 3.6 (90% CI, 1.8–4.9) months. By reviewing the available literature, we identified, however, multiple case reports of patients with HRD-mutated EC with a more pronounced response to PARP-inhibitors. We identified a case of complete response with olaparib in metastatic high-grade serous EC with a somatic *BRIP1*-mutation [60], a case of complete response with olaparib in metastatic endometroid, POLE hypermutated EC with somatic *BRIP1*-, *ATM-*, and *RAD51C*-mutation [61], a case of complete remission with olaparib in recurrent metastatic clear cell EC with somatic *BRCA2*-mutation [62], and a case of partial response with niraparib in a patient with high-grade serous EC with brain metastases and a germline *BRCA1*-mutation [63].

Details concerning the prior treatments and extent of response to PARP-inhibitors in the mentioned patients are summarized in Table 1.

**Table 1:** Clinical data of four patients with *BRIP1-*, *BRCA2-*, or *BRCA1-*mutated EC treated with PARP-inhibitors.

Case study	HRD-mutation	Pretreatment details	Response
Nakamura K. et al. [60]	Somatic *BRIP1*	Debulking surgery followed by first-line carboplatin/paclitaxel and second-line doxorubicin/cisplatin chemotherapy.	Because of persistent para-aortic and left lateral iliac enlarged lymph nodes, treatment with off-label olaparib 300 mg BID was started. 3 months after olaparib initiation, disappearance of all residual swollen lymph nodes in the pelvis was noted on CT-scan. At 9 months of treatment, the patient is in continued CR.
Senguttuvan R. et al. [61]	Somatic *BRIP1*, *ATM*, and *RAD51C*	After debulking surgery, adjuvant chemotherapy with carboplatin/paclitaxel was initiated but was paused several times due to poor performance status and complications. After 3 cycles, recurrent disease was seen with new hepatic metastases, peritoneal carcinomatosis and supraclavicular, retroperitoneal, and pelvic nodal metastasis. The patient was switch to liposomal doxorubicin and received one cycle, but this was discontinued due to severe cutaneous toxicity necessitating hospitalization.	Olaparib 200 mg BID in compassionate use was started with a complete clinical response after 1 year. Olaparib treatment was stopped after 3 years with a currently ongoing CR.
Anzellini D. et al. [62]	Somatic *BRCA2*	Total laparoscopic hysterectomy was performed, and regular follow-up was started. On a follow-up PET-CT, both local recurrence at the vaginal dome and 4 lung metastases were noted. The nodule at the vaginal dome was resected, and systemic therapy with carboplatin/paclitaxel/bevacizumab was started. After 3 cycles, a PET-CT re-evaluation showed that 3 out of 4 lung nodules disappeared, and SBRT of the remaining lung nodule was performed. After the SBRT, bevacizumab maintenance therapy was continued. At the next evaluation, however, further recurrence at the vaginal dome was seen.	Olaparib 300 mg BID was started. After at 5 months of treatment, a PET/CT showed CR. Unfortunately, 3 months later, two cerebral brain metastases in the right fronto-parietal lobe were seen on MRI. Then, stereotactic radiosurgery of the brain metastases was performed while continuing olaparib, at the same dosage as before. 24 months after starting olaparib treatment and 15 months after the end of stereotactic radiotherapy, the patient is in complete remission.
Wang Q. et al. [63]	Germline *BRAC1*	Debulking surgery followed by adjuvant chemotherapy with liposomal doxorubicin/carboplatin for 6 cycles. The patient later development brain metastases, for which she received whole brain radiotherapy in combination with temozolomide with initial improvement of symptoms but intracranial progression shortly after.	Niraparib 200 mg QD was started because of disease progression on MRI accompanied by progressive neurological symptoms. Two month after the start of niraparib, neurological symptoms disappeared. Cranial MRI after 5 months showed PR. Unfortunately, after 9 months of niraparib, neurological symptoms returned with PD on cranial MRI.

SD: stable disease; PD: progressive disease; CR: complete response; PR: partial response; EC: endometrial cancer; PARP: poly(ADP-ribose) polymerase inhibitors: MRI: magnetic resonance imaging.

#### PARP-inhibitors in sarcoma patients with HRD-mutations

As is shown by our case report, HRD might play a role in STS. In the presented case, a complete remission with olaparib was observed for a patient with metastatic high-grade pleomorphic sarcoma with a germline *BRIP1*-mutation. To our knowledge, no previous case of long-term complete remission obtained by the use of PARP-inhibitor in *BRIP1*-mutated sarcomas has been published. The presence of HRD and the use of PARP-inhibitors in sarcomas have, however, been studied before, albeit mostly in a preclinical setting and with variable outcome [64–80].

The most interesting STS subtype in regard of HRD seems to be uterine leiomyosarcoma (uLMS), as growing evidence is supporting the observation that a significant subset of uLMS patients exhibits a HRD phenotype [77–79]. A pan-cancer analysis of germline and somatic *BRCA*-alterations by Jonsson P. et al. [80] even noted that uLMS exhibited the highest rate of homozygous *BRCA2*-deletion, which might indicate a previously unrecognized *BRCA*-dependent cancer type [80]. In both a 2019 case study by Seligson N. et al. [79] and a 2023 case study by Dall G. et al. [81], pathological *BRCA2*-alterations were identified in approximately 10% of the included uLMS cases. In both studies, treatment with off-label olaparib was started if a *BRCA2*-mutation was present with evidence of clinical benefit in all reported cases. Similarly, in a third case study by Pan M. et al. [82], a rapid response to olaparib in a single patient with confirmed *BRCA2*-mutated uLMS was observed. Details concerning the prior treatments and extent of response to olaparib in the mentioned uLMS patients are summarized in Table 2.

**Table 2:** Clinical data of nine patients with *BRCA2*-mutated uterine leiomyosarcoma treated with olaparib [79, 81, 82].

Case study		Number of prior systemic therapies	Pretreatment details	Response to olaparib
1.	Seligson N. et al.	3	Gemcitabine-docetaxel, cisplatin, doxorubicin-ifosfamide and pelvic mass resection and pelvic radiation.	After 3 months of olaparib, diminished FDG-avidity of tumoral lesions on PET-CT was noted, and the patient has exhibited ongoing SD on olaparib for 17 months.
2.	Seligson N. et al.	4	Gemcitabine-docetaxel, doxorubicin, pazopanib, and trabectedin	Initial SD followed by a subclinical progression after 5 months of olaparib. At that moment, trabectedin was reinitiated and added to olaparib therapy. This regimen stabilized the patient’s disease for 15 months before PD.
3.	Seligson N. et al.	2	Intensive chemotherapy was not an option due to comorbidities. The patient was started on pazopanib for 1 year with the addition of everolimus for an additional year because of subclinical tumor progression	After further progression, the patient was initiated on olaparib with ongoing SD after 16 months.
4.	Seligson N. et al.	2	Gemcitabine-docetaxel, ifosfamide-etoposide, resection of pulmonary metastases, and radiation of pulmonary, pelvic, adrenal, and pancreatic metastases	Data are still immature. 10 weeks after initiation of olaparib, the largest abdominal mass had decreased by 62%. No new tumors had appeared over this period, and the patient remained stable 16 weeks after initiating olaparib.
5.	Dall G. et al.	1	First-line anthracycline-base chemotherapy	Imaging showed SD after 2 months of olaparib and then mixed response at 4 months. The patients received radiation to an enlarging pulmonary metastasis, but despite on-going olaparib developed PD within a further month. Single agent carboplatin was added 7 months after starting PARP-inhibitor with an ongoing PR at 19 months post-initiation of olaparib.
6.	Dall G. et al.	4	NA	PR was achieved after 5 months of olaparib, with the addition of localized radiotherapy at 13 months and 19 months of olaparib use because of oligometastatic PD.
7.	Dall G. et al.	1	First-line anthracycline-base chemotherapy with mixed response	PR was observed on FDG-PET after 6 weeks of olaparib with continued SD after 3 months.
8.	Dall G. et al.	5	Doxorubicin, docetaxel-gemcitabine, pazopanib, ifosfamide, and dacarbazine	The patient achieved PR at 4 months, which was sustained until 12 months, before developing evidence of minor progression in a mediastinal lymph node. Cisplatin was added, and after 3 cycles of cisplatin and olaparib combination therapy, a PR was again observed. After 6 cycles of cisplatin in combination with olaparib, a CR was achieved. Later, upon the development of brain metastases, stereotactic radiosurgery was performed while intermittently continuing on single agent olaparib. At 49 months after starting olaparib, the patient is still in metabolic CR (FDG-PET), outside of the brain.
9.	Pan M. et al.	3	Gemcitabine-docetaxel, doxorubicin, and temozolomide regimens	6 weeks after olaparib initiation, major PR was noted on PET-CT with continued SD at 8 months.

SD: stable disease; PD: progressive disease; CR: complete response; PR: partial response; NA: information not available in source article; PARP: poly(ADP-ribose) polymerase inhibitors; FDR: [¹⁸ F]Fluorodeoxyglucose.

Adding onto these preclinical and isolated case findings, an open-label, phase 1b study from the Italian Sarcoma Group with olaparib and trabectedin in advanced and non-resectable bone and soft-tissue sarcomas (TOMAS) was conducted [83]. Trabectedin chemotherapy causes ssDNA and dsDNA breaks, which theoretically can act synergistically with PARP-inhibitors [83]. The TOMAS trial showed manageable toxicities at active dose levels for both drugs with encouraging preliminary data on anti-tumor activity for STS [83]. The study did not demonstrate a better treatment response in the presence of HRD but was underpowered in this regard. The activity in STS is being further assessed in the ongoing TOMAS2 trial [84]. This is a randomized phase 2 study comparing trabectedin alone versus the combination of trabectedin and olaparib (NCT2018-004497-10). Based on preliminary data [84], the trial failed to meet its primary endpoint. Nevertheless, it should be noted that 20% of patients in the trabectedin + olaparib group were treated for over 1 year. With the available information, it currently remains an unanswered question whether or not these long responders have a higher rate of HRD-mutations.

## Discussion and conclusion

We presented a case of metastatic high-grade pleomorphic sarcoma with germline *BRIP1*-mutation who achieved long-term and ongoing complete remission on olaparib. Currently, at 36 months of treatment, the patient remains in oncological remission with an excellent quality of life. Even more so than the extent of the response, the duration of the response to first-line olaparib treatment is noteworthy.

Olaparib and other PARP-inhibitors have already proven their survival benefit and role in clinical practice in the context of metastatic *BRCA*-mutated breast, prostate, and pancreatic cancers and in HRD or platinum-sensitive ovarian cancer [20–23].

In light of the presented case, two important findings were recognized: the significance of *BRIP1* as a potential therapeutic target and the presence of a HRD phenotype in ‘non-traditional’ cancer types.

Similar to *BRCA1*/*2*, *BRIP1* is believed to be an important player in the HR-pathway, which enables a cell to restore dsDNA breaks and maintain genomic stability. Loss-of-function mutations in *BRIP1* can, therefore, promote cancer development. Indeed, studies have identified *BRIP1* as a cancer susceptibility gene [13, 17, 18]. Moreover, the assumption has been made that patients with *BRIP1*-mutated tumors could benefit from PARP-inhibition, which seems logical from a pathophysiological point of view. Clinical data regarding this topic are scarce because of the low number of patients with a confirmed *BRIP1*-mutation included in studies with PARP-inhibitors in non-*BRCA*-mutated HRD cancer. However, the clinical data, which is available, seem to confirm this hypothesis and support the use of PARP-inhibitors in the context of *BRIP1*-mutated breast, ovarian, and prostate cancers [35–37, 45]. Moreover, the rapid and pronounced response to olaparib in the presented case alludes to a broader field of beneficial PARP-inhibitor use, extending to other tumor types, including STS. This is further illustrated by the germline testing of the presented patient’s sister with early-onset breast cancer, which revealed the same *BRIP1* c.1871C>A p.(Ser624*) mutation, suggesting a shared inherited cancer susceptibility gene mutation.

Building onto these findings, we suggest a tumor-agnostic approach for *BRIP1*-mutated cancer, in which the start of a PARP-inhibitor could be considered as a therapeutic option regardless of the tumor type. The identification of a *BRIP1*-mutation can therefore be impactful, especially in cancers with limited treatment options or in patients unable to tolerate more intensive treatment regimens. We see a parallel with neurotrophic tyrosine receptor kinase (NTRK) inhibitors, which are used to treat patients with advanced solid tumors with a NTRK-gene fusion [85]. Treatment with NTRK-inhibitors is tumor agnostic and dependent on the presence of NTRK-fusions only. A similar approach could be followed for cancers with a *BRIP1*- or other HRD-mutation. Such a tumor-agnostic approach requires a strong emphasis on genomic characterization of patients with (metastatic) cancer.

This article has both strengths and limitations. The diagnosis of metastatic disease was confirmed by resection of a metastatic lesion, and both a central pathology review and central genomic assessment were performed because of clinical trial participation.

To the best of our knowledge, no previous case of long-term complete remission obtained by the use of PARP-inhibitor in *BRIP1*-mutated sarcomas has been published. Nevertheless, the presence of HRD and the use of PARP-inhibitors in sarcomas have been studied earlier, with some cases of clinical remission on olaparib in *BRCA2*-mutated uLMS [79, 81, 82]. Since the treatment options for metastatic high-grade pleomorphic sarcoma are limited, this new therapeutic route is of great interest and warrants further investigation.

Furthermore, an attempt was made to provide a comprehensive overview of the available literature in order to support our recommendations. As this is a single case, our findings need to be interpretated with caution. For reasons stated in the material and methods section, a narrative review rather than a systematic review was performed, and, therefore, potential relevant information could have been missed.

Furthermore, no conclusion about different loss-of-function mutations in *BRIP1* in relation to potential impact on response to PARP-inhibition could be made. We identified this as a topic for further research.

Finally, the provided insights cannot be directly translated into clinical practice, as a comprehensive genomic assessment is not routinely performed in STS and was, in this case only possible by participation in the BALLETT study. The benefit and feasibility of the clinical implementation of this approach is currently examined within the BSMO-Precision Framework, which results are expected soon [10].

## Supplementary Material



## Data Availability

No dataset was used, and no any statistical analysis was performed. The anonymized patient’s data are presented in this article. Additional information may be requested from the corresponding author.
