# Supplementary material for: Complete remission with olaparib in BRIP1-mutated metastatic high-grade pleomorphic sarcoma: case study and literature review – an example of a genomic profiling-based tumor treatment, in a cancer type with high unmet clinical need
Source: Acta Oncol. 2025 Sep 23;64:43374. doi: 10.2340/1651-226X.2025.43374 (PMC12476058; doi:10.2340/1651-226X.2025.43374)
Supplement: Supplementary file 1 [file AO-64-43374-s1.pdf]

Appendix 1 : *BRIP1* and homologous recombination deficiency

Human DNA typically takes the form of a double helix, comprised of two homologous DNA strands. During DNA unwinding, which is necessary in the process of DNA replication, a part of this structural integrity gets temporally lost and both single-strand (ssDNA) and double-strand (dsDNA) breaks can occur. Likewise, exposure to DNA damaging agents such as ionizing radiation or cross-linking agents can also induce DNA breaks[1]. These DNA breaks constitute a severe threat for the integrity of the genome and can be important drivers in carcinogenesis[2]. Fortunately, multiple pathways for the repair of ssDNA and dsDNA breaks have evolved in order to mitigate this risk[2]. dsDNA breaks can either be repaired by homologous recombination (HR), which uses the undamaged sister chromatid as a template and is therefore mostly error-free or by non-homologous end-joining (NHEJ), being more error-prone[1].

When a dsDNA break occurs a complex DNA damage response is triggered, involving sensors that can detect broken ends; effectors that execute repair; mediators that facilitate interactions between sensors and effectors; and checkpoints that delay the cell cycle during the DNA repair process[1]. Important players in this HR-pathway are *BRCA1* and *BRCA2*, as well as *PALB2* and *BRIP1*.

BRCA1 binds to dsDNA breaks and is thought to function as a platform on which initial DNA damage sensing proteins assemble. Furthermore, complexes involving *BRCA1* are responsible for 5′-end resection of dsDNA breaks, which is an essential step before restorative DNA synthesis can start[3].

BRCA2 exerts its function downstream in the HR-cascade by recruiting effectors that are responsible for the actual DNA repair. *PALB2* binds directly to both *BRCA1* and *BRCA2* and thereby provides a physical link between the two proteins, facilitating their function[1].

BRCA1 interacting protein (*BRIP1*), also known as *BRCA1* interacting C-terminal helicase (BACH1) or Fanconi anemia J (FANCJ) helicase, is a more recently discovered tumor suppressor[1,4,5]. *BRIP1* is thought to execute his function by unwinding forked duplex DNA substrates at the site of the dsDNA breaks[4], thereby enabling the *BRCA2-PALB2* complex to start homologous recombination of the newly unwinded stalled replication forks[6]. Moreover, *BRIP1* is believed to facilitate smooth progression of the newly synthesized DNA strands by resolving alternate DNA structures such as G-quadruplexes that can hinder replication[7,8]. Lastly, the direct interaction of *BRIP1* with *BRCA1* seems to be necessary for checkpoint control, which limits the DNA damage tolerance of the cell[1].

An overview of this DNA damage repair pathway is presented in figure 1.

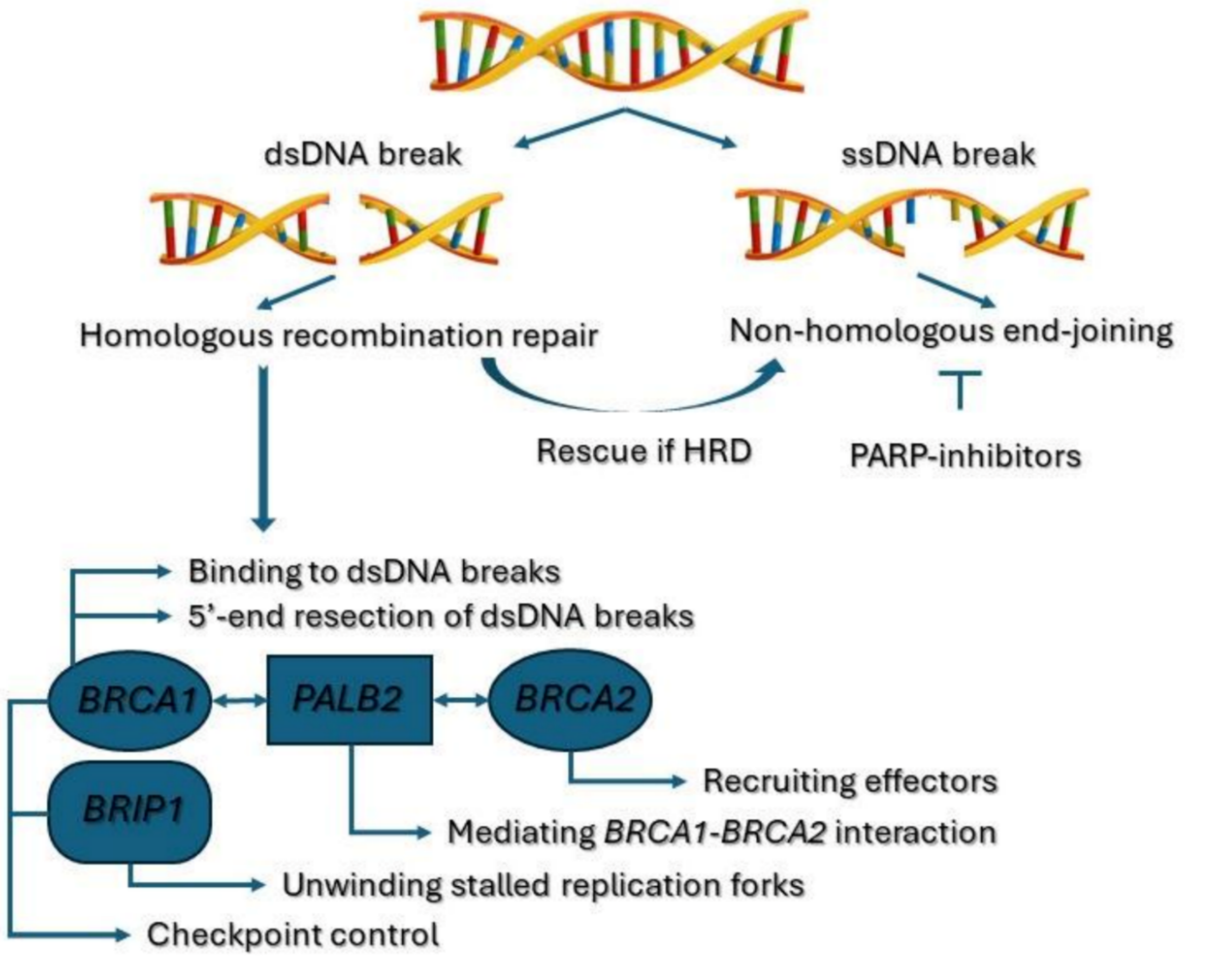

Figure 1: Role of *BRCA1, PALB2, BRCA2* and *BRIP1* in the homologous recombination pathway

[1] Roy R, Chun J, Powell SN. BRCA1 and BRCA2: different roles in a common pathway of genome protection. Nat Rev Cancer 2012;12:68–78.
[2] Stewart MD, Merino Vega D, Arend RC, Baden JF, Barbash O, Beaubier N, et al. Homologous recombination deficiency: concepts, definitions, and assays. Oncologist 2022;27:167–74.
[3] Symington LS. End resection at double-strand breaks: mechanism and regulation. Cold Spring Harb Perspect Biol 2014;6:a016436.
[4] Cantor SB, Guillemette S. Hereditary breast cancer and the BRCA1-associated FANCJ/BACH1/BRIP1. Future Oncol 2011;7:253–61. https://doi.org/10.2217/fon.10.191.
[5] Cantor SB, Bell DW, Ganesan S, Kass EM, Drapkin R, Grossman S, et al. BACH1, a Novel Helicase-like Protein, Interacts Directly with BRCA1 and Contributes to Its DNA Repair Function. Cell 2001;105:149–60. https://doi.org/10.1016/S0092-8674(01)00304-X.
[6] Peake JD, Noguchi E. Fanconi anemia: current insights regarding epidemiology, cancer, and DNA repair. Hum Genet 2022;141:1811–36. https://doi.org/10.1007/s00439-022-02462-9.
[7] Brosh Jr RM, Cantor SB. Molecular and cellular functions of the FANCJ DNA helicase defective in cancer and in Fanconi anemia. Front Genet 2014;5:372.
[8] Wang W. Emergence of a DNA-damage response network consisting of Fanconi anaemia and BRCA proteins. Nat Rev Genet 2007;8:735–48. https://doi.org/10.1038/nrg2159.

Appendix 2: Authorized indications for different PARP-inhibitors by the European Medical Agency

	**High-grade ovarian, fallopian and peritoneal cancer**	**Breast cancer**	**Pancreatic cancer**	**Prostate cancer**
**Olaparib (Lynparza)** [9]	- Platinum-sensitive relapse (regardless of *BRCA* status)<br>- Maintenance monotherapy in platinum-sensitive disease with known germline or somatic *BRCA1/2* mutation<br>- Maintenance combination therapy with bevacizumab in platinum-sensitive disease with HRD	- Monotherapy or in combination with endocrine therapy for the adjuvant treatment of adult patients with germline *BRCA1/2*-mutations who have HER2-negative, high risk early breast cancer previously treated with neoadjuvant or adjuvant chemotherapy<br>- Monotherapy for the treatment of adult patients with germline *BRCA1/2*-mutations, who have HER2 negative locally advanced or metastatic breast cancer.<br>- Patients should have previously been treated with an anthracycline and a taxane in the (neo)adjuvant or metastatic setting. Patients with hormone receptor positive breast cancer should also have progressed on or after prior endocrine therapy, or be considered unsuitable for endocrine therapy.	- Maintenance treatment of patients with germline BRCA1/2-mutations who have metastatic adenocarcinoma of the pancreas and have not progressed after a minimum of 16 weeks of platinum treatment within a first-line chemotherapy regimen.	- Monotherapy for the treatment of patients with metastatic castration-resistant prostate cancer and BRCA1/2-mutations (germline and/or somatic) who have progressed following prior therapy that included a new hormonal agent.<br>- In combination with abiraterone and prednisone or prednisolone for the treatment of adult patients with mCRPC in whom chemotherapy is not clinically indicated.
**Niraparib (Zejula)** [10]	- Platinum-sensitive relapse (regardless of *BRCA* status)<br>- Maintenance monotherapy in platinum-sensitive disease (regardless of BRCA status)	/	/	/
**Talazoparib (Talzenna)**[11]	/	- Monotherapy for the treatment of adult patients with germline *BRCA1/2*-mutations, who have HER2-negative locally advanced or metastatic breast cancer.<br>Patients should have previously been treated with an anthracycline and a taxane in the (neo)adjuvant or metastatic setting. Patients	/	/

		with hormone receptor positive breast cancer should also have progressed on or after prior endocrine therapy, or be considered unsuitable for endocrine therapy.		
**Rucaparib (Rubraca)** [12]	- Platinum-sensitive relapse (regardless of BRCA status)	/	/	/

[9] European Medicines Agency. Lynparza EMA authorisation 2024. https://www.ema.europa.eu/en/medicines/human/EPAR/lynparza (accessed January 15, 2024).

[10] European Medicines Agency. Zejula EMA authorisation 2024. https://www.ema.europa.eu/en/medicines/human/EPAR/zejula (accessed January 15, 2024).

[11] European Medicines Agency. Talzenna EMA authorisation 2021. https://www.ema.europa.eu/en/medicines/human/EPAR/talzenna (accessed January 15, 2024).

[12] European Medicines Agency. Rubraca EMA authorisation 2023. https://www.ema.europa.eu/en/medicines/human/EPAR/rubraca (accessed January 15, 2024).
